# Numerical Investigation of Mixed Convection and Entropy Generation in a Wavy-Walled Cavity Filled with Nanofluid and Involving a Rotating Cylinder

**DOI:** 10.3390/e20090664

**Published:** 2018-09-03

**Authors:** Ammar I. Alsabery, Muneer A. Ismael, Ali J. Chamkha, Ishak Hashim

**Affiliations:** 1 Department of Refrigeration & Air-conditioning Technical Engineering, College of Technical Engineering, The Islamic University, Najaf 54001, Iraq; 2 School of Mathematical Sciences, Faculty of Science & Technology, Universiti Kebangsaan Malaysia, Bangi Selangor 43600, Malaysia; 3 Department of Mechanical Engineering, Engineering College, University of Basrah, Basrah 61004, Iraq; 4 Department of Mechanical Engineering, Prince Sultan Endowment for Energy and the Environment, Prince Mohammad Bin Fahd University, Al-Khobar 31952, Saudi Arabia; 5 RAK Research and Innovation Center, American University of Ras Al Khaimah, P.O. Box 10021, Ras Al Khaimah 86416, UAE

**Keywords:** entropy generation, mixed convection, wavy cavity, rotating solid cylinder, heat source

## Abstract

This numerical study considers the mixed convection and the inherent entropy generated in Al${}_{2}$O${}_{3}$–water nanofluid filling a cavity containing a rotating conductive cylinder. The vertical walls of the cavity are wavy and are cooled isothermally. The horizontal walls are thermally insulated, except for a heat source segment located at the bottom wall. The dimensionless governing equations subject to the selected boundary conditions are solved numerically using the Galerkin finite-element method. The study is accomplished by inspecting different ranges of the physical and geometrical parameters, namely, the Rayleigh number ($10^{3}\leq Ra\leq 10^{6}$), angular rotational velocity (0≤Ω≤750), number of undulations (0≤N≤4), volume fraction of Al${}_{2}$O${}_{3}$ nanoparticles (0≤ϕ≤0.04), and the length of the heat source (0.2≤H≤0.8). The results show that the rotation of the cylinder boosts the rate of heat exchange when the Rayleigh number is less than $5\times 10^{5}$. The number of undulations affects the average Nusselt number for a still cylinder. The rate of heat exchange increases with the volume fraction of the Al${}_{2}$O${}_{3}$ nanoparticles and the length of the heater segment.

## 1. Introduction

Although natural convection contributes vitally in releasing or adding energy from enclosures, mixed convection might be more appropriate in such a task. Mixed convection is often regarded for efficient heat transfer removal or control in cooling of electric and electronic systems, lubrication [1], emergency cooling systems of nuclear reactors, etc. Moving surfaces are considered a common mechanism to introduce the mixed convection in enclosures. Moving surfaces may be lid-driven walls or rotating bodies inside the enclosure. Other strategies can contribute in enhancing the process of heat removal such as the use of nanofluids and increasing the surface area. Nanofluids are achieved by adding nanoparticles (∼100 nm-diameter-sized) of different properties, metal or ceramic to the base fluid that has a low thermal conductivity [2]. Increasing of the surface area can be carried out by corrugating some isothermal walls of the enclosures [3]. According to the second law of thermodynamics, entropy inevitably accompanies the process of heat transfer. Thus, to maintain a maximum use of energy, the analysis of entropy generation in an enclosure has received a considerable attention in the literature. As a mean of explaining the role of the present paper, the topics mentioned above will be reviewed sequentially in the following survey.

The mixed convection obtained from the lid-driven mechanism is related to the current subject in terms of the movement of the solid walls. The base of the lid-driven cavity may depend on the early study of Torrance et al. [4], who revealed a distinct role of buoyant force at a Grashof number $Gr=\pm 10^{6}$ for a cavity with a lid-driven horizontal wall. Thereafter, many studies have been devoted for this topic. Iwatsu et al. [5] figured the combined convection in 3D lid-driven cavity, then in another study, they extended the parameters but for a 2D cavity [6]. Abu-Nada and Chamkha [7] evaluated the role of the lid-driven mechanism on the combined convection in a cavity filled with a CuO–water nanofluid. Al-Amiri et al. [8] documented the combined thermal and mass transport in a square lid-driven cavity. Ismael et al. [9] have reported the impact of partial slip accompanying the lid-driven wall in high temperature applications. Ismael [10] has considered the effect of combined convection in a cavity of arc-shaped lid-driven wall. Alsabery et al. [11] have considered the Brownian motion and the thermophoresis effect to investigate conjugate combined convection in a double lid-driven cavity filled with ${\mathrm{Al}}_{2}O_{3}$–water nanofluid and including a solid inner body. Many other lid-driven geometries were reviewed in [10].

To avoid the technical problems arising with moving cavity walls, a rotating circular cylinder is included. This strategy has the possibility of changing the position of rotation easily. Fu et al. [12] considered an isothermal rotating surface to enhance the natural convection in enclosures. Yoon et al. [13] investigated the impact of the position of a hot circular cylinder on free convection in a square cavity at a very high Rayleigh number ($Ra=10^{7}$). Costa and Raimundo [14] studied the impact of imposing a rotated cylinder on the process of heat exchange taking into account the conductive heat transfer and the advection of enthalpy in the cylinder. Chatterjee et al. [15] included the effect of an externally applied magnetic field into a cavity involving a cylinder rotating at the cavity center. Liao and Lin [16] have confirmed the feasibility of the immersed-boundary approach for the sake of studying free convection in a square cavity involving a concentric solid cylinder. Roslan et al. [17] used COMSOL Multiphysics to simulate the combined convection of nanofluids filling a cavity heated and cooled from sides and involving a centered rotating cylinder. They deduced that a slower rotation with a mild cylinder size of a circular cylinder gives a maximal heat exchange. Liao and Lin [18] considered the impact of a varying fluid type on the free convection instability in a square cavity involving a hot cylinder. Wang et al. [19] carried out a numerical investigation of combined convection in a triangular cavity filled with a SiC-EG nanofluid and including a circular rotating cylinder. Their results clearly showed a significant dependence of the the thermal performance with the rotation sense. Chamkha et al. [20], Selimefendigil et al. [21], and Ismael et al. [22] published a series of studies regarding the effect of rotating cylinder inside composite cavities.

Adjlout et al. [23], Varol and Oztop [24], Oztop et al. [25], Nasrin [26], and Mekroussi et al. [27] have investigated vertical and shallow cavities of wavy walls. Nanofluids enhancement is also evaluated inside wavy enclosures; Nasrin et al. [28] considered an enclosure filled with a CuO–water nanofluid composed of triangular-wavy wall and moving horizontal walls. Abu-Nada and Chamkha [7] dealt with a CuO–water nanofluid filled in a lid-driven cavity with a wavy bottom wall of single undulation. They inspected the combined convection within a nanofluid. Hatami et al. [29] considered the free convection of a nanofluid inside a novel circular cavity with a wavy wall. The response surface methodology was followed to optimize the geometry of the cavity. They figured out that the amplitude of the wavy wall is more effective than the number of undulations. Sheremet et al. [30] employed Buongiorno’s model to discuss the unsteady free convection of a nanofluid filled in a cavity with a wavy wall and subjected to a constant magnetic field. It is worth mentioning that Xiao et al. [31] have proposed a comprehensive model to predict the convective heat transfer in nanofluids based on the Brownian motion and the fractal distribution of nanoparticle sizes. This model revealed the physical explanation of the decrease of convective heat transfer with the increase in the nanoparticles size. As a latest development of nanofluid fractal model, Xiao et al. [32,33] have promoted their previous model by excluding any empirical constant.

For the sake of energy saving, it is important to utilize the maximum available energy from thermal systems. Entropy generation related with the thermodynamic constraints is followed as a route to evaluate the performance. Various reasons behind entropy generation in applied thermal engineering are listed by Bejan [34,35,36]. Mahmud and Fraser [37] have examined the nature of entropy generation in the heat exchange within a wavy-walled cavity. They found that at higher Rayleigh numbers, the fluid friction mostly causes the rise of entropy. Bouabid et al. [38] considered the unsteady free convection and the accompanying entropy generation due to various aspect ratios and tilt angles of a rectangular cavity. Ilis et al. [39] discussed the generation of entropy due to natural convection in a rectangular enclosure heated vertically. They studied the impact of various aspect ratios with keeping the original space of the cavity constant. They predicted that when the buoyancy force is predominant, the friction-related entropy generation increases with the increase of the aspect ratio. Cheng and Liang [40] emphasized that for the sake of optimizing any thermodynamic system, minimizing the rate of entropy generation is a major condition. Mamourian et al. [41] have adopted the analysis of the Taguchi method to optimize the mixed convection and entropy generation in a wavy surface square cavity. They demonstrated a decrease in the Nusselt number and the entropy generation as the wavelength of the wavy surface increases. Esfahani et al. [42] investigated the generation of entropy for a copper–water nanofluid in a horizontal wavy channel. They observed that the increase of entropy generation with the Reynolds number becomes significant with increasing the wave amplitude. Sheremet et al. [43] studied the natural convection of a copper–water nanofluid filled in a gap between a square cavity and an isothermal square solid insertion. Increasing functions of entropy generation with the size of the solid insertion and the volume fraction of the nanoparticles were declared. However, the following citations are examples for the case of analyzing the thermodynamic irreversibility in nanofluids; Kashani et al. [44]; Cho et al. [45]; Ting et al. [46]; Ismael et al. [47]; Cho et al. [48]; Chamkha et al. [49]; Chamkha et al. [50]; Rashidi et al. [51]; Qasim et al. [52]; Darbari et al. [53] and Kefayati and Tang [54]. Very recently, Alsabery et al. [55] have studied the entropy generation inside a porous cavity with wavy walls including a rotating cylinder. They showed a decrease in the fluid friction irreversibility with increasing the undulations of the wavy walls.

Based on the above survey, it is acknowledged now that there are few comprehensive works that study the nanofluid flow, heat exchange and the entropy generation in a wavy-walled cavity including a conductive rotating cylinder. Thus, the current paper investigates the main aspects of such a problem under the assumption of laminar 2D flow.

## 2. Mathematical Formulation

**Figure 1:**
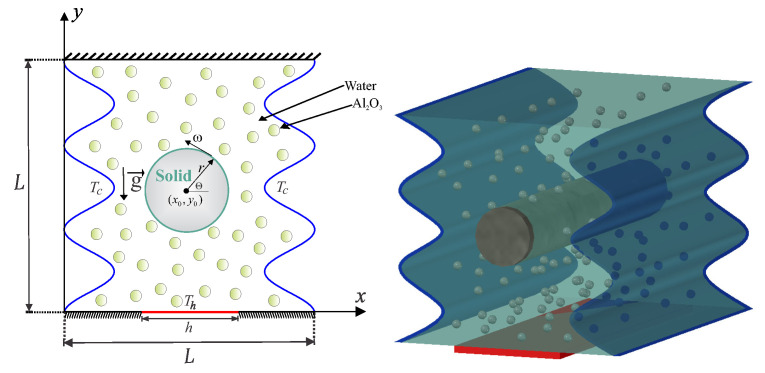
Schematic diagram of the physical model together with the rotating conductive cylinder and the coordinate system.

Consider the steady mixed convection heat transfer in a wavy-wall cavity with length *L* and continuing a rotating solid cylinder within the center with radius *r*, is illustrated in Figure 1. The vertical walls of the cavity are wavy and cooled isothermally. The horizontal walls are thermally insulated, except a heat source segment located at the bottom wall with length *h*. The boundaries of the domain are taken to be impermeable, the space between the wavy cavity and the rotating cylinder is filled with a water–Al${}_{2}$O${}_{3}$ nanofluid. The Boussinesq approximation is applicable. With the above-mentioned assumptions, the continuity, momentum and energy equations for the Newtonian fluid, laminar and steady state flow can be written as follows [56,57]:

(1) $$\begin{array}{l} \nabla \cdot v=0, \end{array}$$

(2) $$\begin{array}{l} \rho_{nf}v\cdot \nabla v=-\nabla p+\nabla \cdot \left(\mu_{nf}\nabla v\right)+{\left(\rho \beta \right)}_{nf}\left(T-T_{c}\right)\vec{g}, \end{array}$$

(3) $$\begin{array}{l} v\cdot \nabla T=\alpha_{nf}\nabla^{2}T. \end{array}$$

The energy equation of the rotating conductive cylinder is

(4) $$\nabla^{2}T_{s}=0,$$

where $\vec{g}$ signifies the gravitational acceleration vector, v describes the velocity vector, $\rho_{nf}$ represents the density of the nanofluid and $\mu_{nf}$ shows the dynamic viscosity of the nanofluid.

Now we describe the thermophysical properties of the nanofluid as the following [58]:

(5) $${\left(\rho C_{p}\right)}_{nf}=\left(1-\phi \right){\left(\rho C_{p}\right)}_{f}+\phi {\left(\rho C_{p}\right)}_{p}.$$

(6) $$\begin{array}{c} \alpha_{nf}=\frac{k_{nf}}{{\left(\rho C_{p}\right)}_{nf}}. \end{array}$$

(7) $$\begin{array}{c} \rho_{nf}=\left(1-\phi \right)\rho_{f}+\phi \rho_{p}. \end{array}$$

(8) $$\begin{array}{c} {\left(\rho \beta \right)}_{nf}=\left(1-\phi \right){\left(\rho \beta \right)}_{f}+\phi {\left(\rho \beta \right)}_{p}. \end{array}$$

The dynamic viscosity and thermal conductivity ratios of nanofluids (water–Al${}_{2}$O${}_{3}$) with 33 nm particle-size have been adapted according to [59]:

(9) $$\frac{\mu_{nf}}{\mu_{f}}=1/\left(1-34.87{\left(d_{p}/d_{f}\right)}^{-0.3}\phi^{1.03}\right),$$

(10) knfkf=1+4.4ReB0.4Pr0.66TTfr10kpkf0.03ϕ0.66,

where ReB is explained as [59]:

(11) ReB=ρfuBdpμf,uB=2kbTπμfdp2,

and $k_{b}=1.380648\times 10^{-23}\left(J/K\right)$ refers to Boltzmann constant. $l_{f}=0.17$ nm reflects fluid particles mean path. $d_{f}$ denotes the water molecular diameter, as given by [59]:

(12) $$d_{f}=\frac{6M}{N\pi \rho_{f}},$$

where *M* stands for base fluid molecular weight, *N* is Avogadro number, and $\rho_{f}$ idenotes base fluid density at standard temperature (310 K). With water as the based fluid, the value of $d_{f}$ is obtained as the following [59]:

(13) $$d_{f}={\left(\frac{6\times 0.01801528}{6.022\times 10^{23}\times \pi \times 998.26}\right)}^{1/3}=3.85\times 10^{-10}\;m.$$

The following presents non-dimensional variables applied in this study:

(14) $$\begin{array}{c} X=\frac{x}{L},\;Y=\frac{y}{L},\;V=\frac{vL}{\alpha_{f}},\;\theta =\frac{T_{nf}-T_{c}}{T_{h}-T_{c}},\;\theta_{s}=\frac{T_{s}-T_{c}}{T_{h}-T_{c}},\;R=\frac{r}{L},\\ \mathrm{Pr}=\frac{\nu_{f}}{\alpha_{f}},\;Ra=\frac{g\beta_{f}\left(T_{h}-T_{c}\right)L^{3}}{\nu_{f}\alpha_{f}},\;P=\frac{pL^{2}}{\rho_{f}\alpha_{f}^{2}},\;\Omega =\frac{\omega L^{2}}{\alpha_{f}},\;H=\frac{h}{L}. \end{array}$$

Equations (1)–(4), on using Equation (14), now become:

(15) $$\begin{array}{c} \nabla \cdot V=0, \end{array}$$

(16) $$\begin{array}{c} V\cdot \nabla V=-\nabla P+\mathrm{Pr}\frac{\rho_{f}}{\rho_{nf}}\frac{\mu_{nf}}{\mu_{f}}\nabla^{2}V+\frac{{\left(\rho \beta \right)}_{nf}}{\rho_{nf}\beta_{f}}Ra\mathrm{Pr}\theta , \end{array}$$

(17) $$\begin{array}{c} V\cdot \nabla \theta =\frac{{\left(\rho C_{p}\right)}_{f}}{{\left(\rho C_{p}\right)}_{nf}}\frac{k_{nf}}{k_{f}}\nabla^{2}\theta , \end{array}$$

(18) $$\begin{array}{c} \nabla^{2}\theta_{s}=0, \end{array}$$

where V is the dimensionless velocity vector (U,V). The dimensionless boundary conditions corresponding to Equations (15)–(18) are given by:

$$\begin{array}{c} \mathrm{On}\mathrm{the}\mathrm{heated}\mathrm{part}\mathrm{of}\mathrm{the}\mathrm{bottom}\mathrm{horizontal}\mathrm{wall}: \end{array}$$

(19) $$\begin{array}{c} U=V=0,\;\;\theta =1,\;\;Y=0,\;\;(1-H)/2\leq X\leq (1+H)/2, \end{array}$$

$$\begin{array}{c} \mathrm{On}\mathrm{the}\mathrm{adiabatic}\mathrm{parts}\mathrm{of}\mathrm{the}\mathrm{bottom}\mathrm{wall}: \end{array}$$

(20) $$\begin{array}{c} U=V=0,\;\;\frac{\partial \theta }{\partial Y}=0,\;\;Y=0,\;\;0\leq X\leq \left(1-H\right)/2\;\;\mathrm{and}\;\;\left(1+H\right)/2\leq X\leq 1, \end{array}$$

$$\begin{array}{c} \mathrm{On}\mathrm{the}\mathrm{left}\mathrm{vertical}\mathrm{wavy}\mathrm{wall}: \end{array}$$

(21) $$\begin{array}{c} U=V=0,\;\;\theta =0,\;\;1-A(1-\cos(2N\pi X)),\;\;0\leq Y\leq 1, \end{array}$$

$$\begin{array}{c} \mathrm{On}\mathrm{the}\mathrm{right}\mathrm{vertical}\mathrm{wavy}\mathrm{wall}: \end{array}$$

(22) $$\begin{array}{c} U=V=0,\;\;\theta =0,\;\;A(1-\cos(2N\pi X)),\;\;0\leq Y\leq 1, \end{array}$$

$$\begin{array}{c} \mathrm{On}\mathrm{the}\mathrm{adiabatic}\mathrm{top}\mathrm{wall}: \end{array}$$

(23) $$\begin{array}{c} U=V=0,\;\;\frac{\partial \theta }{\partial Y}=0,\;\;0\leq X\leq 1,\;\;Y=1, \end{array}$$

(24) $$\begin{array}{c} \theta =\theta_{s},\;\;\mathrm{at}\mathrm{the}\mathrm{outer}\mathrm{solid}\mathrm{cylinder}\mathrm{surface}, \end{array}$$

(25) $$\begin{array}{c} U=-\Omega \left(Y-Y_{0}\right),\;V=\Omega \left(X-X_{0}\right),\;\;\frac{\partial \theta }{\partial n}=K_{r}\frac{\partial \theta_{s}}{\partial n}, \end{array}$$

where $K_{r}=k_{s}/k_{nf}$ is the thermal conductivity ratio over the surface of the rotating conductive cylinder, the absolute velocity of the considered domain can be calculated as [14]:

(26) |V|=|Ω|R.

Following Costa and Raimundo [14], we introduce the modified form of the Richardson number, signifying the relative importance of the natural and forced convection:

(27) $$Ri=\left(Ra/\mathrm{Pr}\right)/Re^{2},$$

where Re is the Reynolds number. For the current numerical work and according to the dimensionless strategy, this parameter can be written as [14]:

(28) $$Ri=\frac{Ra\cdot \mathrm{Pr}}{4\Omega^{2}\cdot R^{4}},$$

for Ω≠0 and R≠0.

The local Nusselt number examines for heated part of the bottom horizontal wall as:

(29) $$Nu_{nf}=-\frac{k_{nf}}{k_{f}}{\left(\frac{\partial \theta }{\partial Y}\right)}_{Y=0}.$$

Lastly, the average Nusselt number is calculated at the heated part of the bottom horizontal wall of the cavity as:

(30) $$\begin{array}{c} {\bar{Nu}}_{nf}=\int_{\frac{1-H}{2}}^{\frac{1+H}{2}}Nu_{nf}\;dX. \end{array}$$

The entropy generation relation is given by [39,57]:

(31) $$\begin{array}{c} S=\frac{k_{nf}}{T_{0}^{2}}\left[{\left(\frac{\partial T}{\partial x}\right)}^{2}+{\left(\frac{\partial T}{\partial y}\right)}^{2}\right]+\frac{\mu_{nf}}{T_{0}}\left[2{\left(\frac{\partial u}{\partial x}\right)}^{2}+2{\left(\frac{\partial v}{\partial y}\right)}^{2}+{\left(\frac{\partial u}{\partial x}+\frac{\partial v}{\partial x}\right)}^{2}\right]. \end{array}$$

In dimensionless form, the local entropy generation can be expressed as:

(32) $$\begin{array}{c} S_{\mathrm{GEN}}=\frac{k_{nf}}{k_{f}}\left[{\left(\frac{\partial \theta }{\partial X}\right)}^{2}+{\left(\frac{\partial \theta }{\partial Y}\right)}^{2}\right]\\ \;\;+\frac{\mu_{nf}}{\mu_{f}}N_{\mu }\left\{2\left[{\left(\frac{\partial U}{\partial X}\right)}^{2}+{\left(\frac{\partial V}{\partial Y}\right)}^{2}\right]+{\left(\frac{\partial^{2}U}{\partial Y^{2}}+\frac{\partial^{2}V}{\partial X^{2}}\right)}^{2}\right\}, \end{array}$$

where, $N_{\mu }$ is the irreversibility distribution ratio [39,57]:

(33) $$\begin{array}{c} N_{\mu }=\frac{\mu_{f}T_{0}}{k_{f}}{\left(\frac{\alpha_{f}}{L(\Delta T)}\right)}^{2}, \end{array}$$

and $S_{\mathrm{GEN}}$ shows the dimensionless entropy generation rate:

(34) $$\begin{array}{c} S_{\mathrm{GEN}}=S_{\mathrm{gen}}\frac{T_{0}^{2}L^{2}}{k_{f}{\left(\Delta T\right)}^{2}}. \end{array}$$

The terms of Equation (33) can be separated according to the following form:

(35) $$\begin{array}{c} S_{\mathrm{GEN}}=S_{\theta }+S_{\Psi }, \end{array}$$

where $S_{\theta }$ and $S_{\Psi }$ are the entropy generation due to heat transfer irreversibility (HTI) and the fluid friction irreversibility (FFI), respectively.

(36) $$\begin{array}{cc} & S_{\theta }=\frac{k_{nf}}{k_{f}}\left[{\left(\frac{\partial \theta }{\partial X}\right)}^{2}+{\left(\frac{\partial \theta }{\partial Y}\right)}^{2}\right], \end{array}$$

(37) $$\begin{array}{c} S_{\Psi }=\frac{\mu_{nf}}{\mu_{f}}N_{\mu }\left\{2\left[{\left(\frac{\partial U}{\partial X}\right)}^{2}+{\left(\frac{\partial V}{\partial Y}\right)}^{2}\right]+{\left(\frac{\partial^{2}U}{\partial Y^{2}}+\frac{\partial^{2}V}{\partial X^{2}}\right)}^{2}\right\}. \end{array}$$

The global entropy generation (GEG) is obtained by integrating Equation (36) over the domain

(38) $$\begin{array}{c} \mathrm{GEG}=\int S_{\mathrm{GEN}}\;dXdY=\int S_{\theta }\;dXdY+\int S_{\Psi }\;dXdY. \end{array}$$

The Bejan number Be defined as:

(39) $$\begin{array}{c} Be=\frac{\int S_{\theta }\;dXdY}{\int S_{\mathrm{GEN}}\;dXdY}. \end{array}$$

When Be>0.5, the HTI is the dominant, while when Be<0.5, the FFI is the dominant.

## 3. Numerical Method and Verification

**Figure 2:**
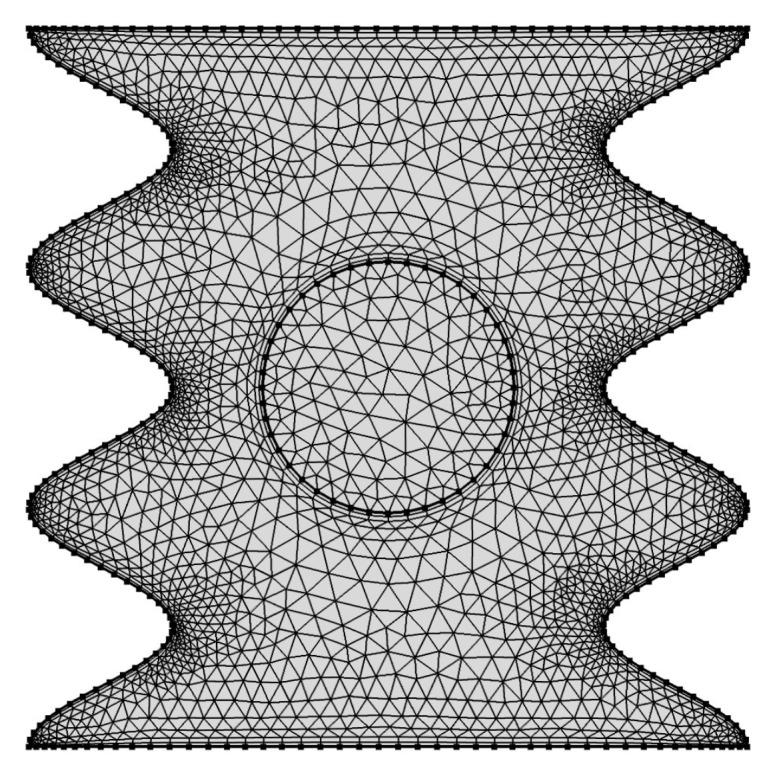
Grid-points distribution for grid size of G6 (27,151 elements).

The dimensionless governing Equations (15)–(18) subject to the selected boundary conditions (19)–(25) are solved with the Galerkin weighted residual finite-element method. The computational domain is discretized into triangular elements as shown in Figure 2. Triangular Lagrange finite elements of different orders are used for each of the flow variables within the computational domain. Residuals for each conservation equation are obtained by substituting the approximations into the governing equations. To simplify the nonlinear terms in the momentum equations, a Newton–Raphson iteration algorithm was used. The convergence of the solution is assumed when the relative error for each of the variables satisfies the following convergence criteria:

$$\begin{array}{c} \left|\frac{\Gamma^{i+1}-\Gamma^{i}}{\Gamma^{i+1}}\right|\leq \eta , \end{array}$$

where *i* denotes the iteration number and η represents the convergence criterion. In the current work, the setting of convergence criterion was done at $\eta =10^{-6}$.

We have employed grids with various sizes to ensure that the present numerical solution is independent on the grid size for the numerical domain, different grid sizes are used to calculate the minimum strength of the flow circulation ($\Psi_{\min}$), average Nusselt number (${\bar{Nu}}_{nf}$) and Bejan number (Be) for the case of Ω=250, N=3, $Ra=10^{5}$, ϕ=0.02 and H=0.5. The results are shown in Table 1 which indicate insignificant differences for the G6 grids and above. Therefore, the G6 uniform grid is employed for all computations in this paper.

**Figure 3:**
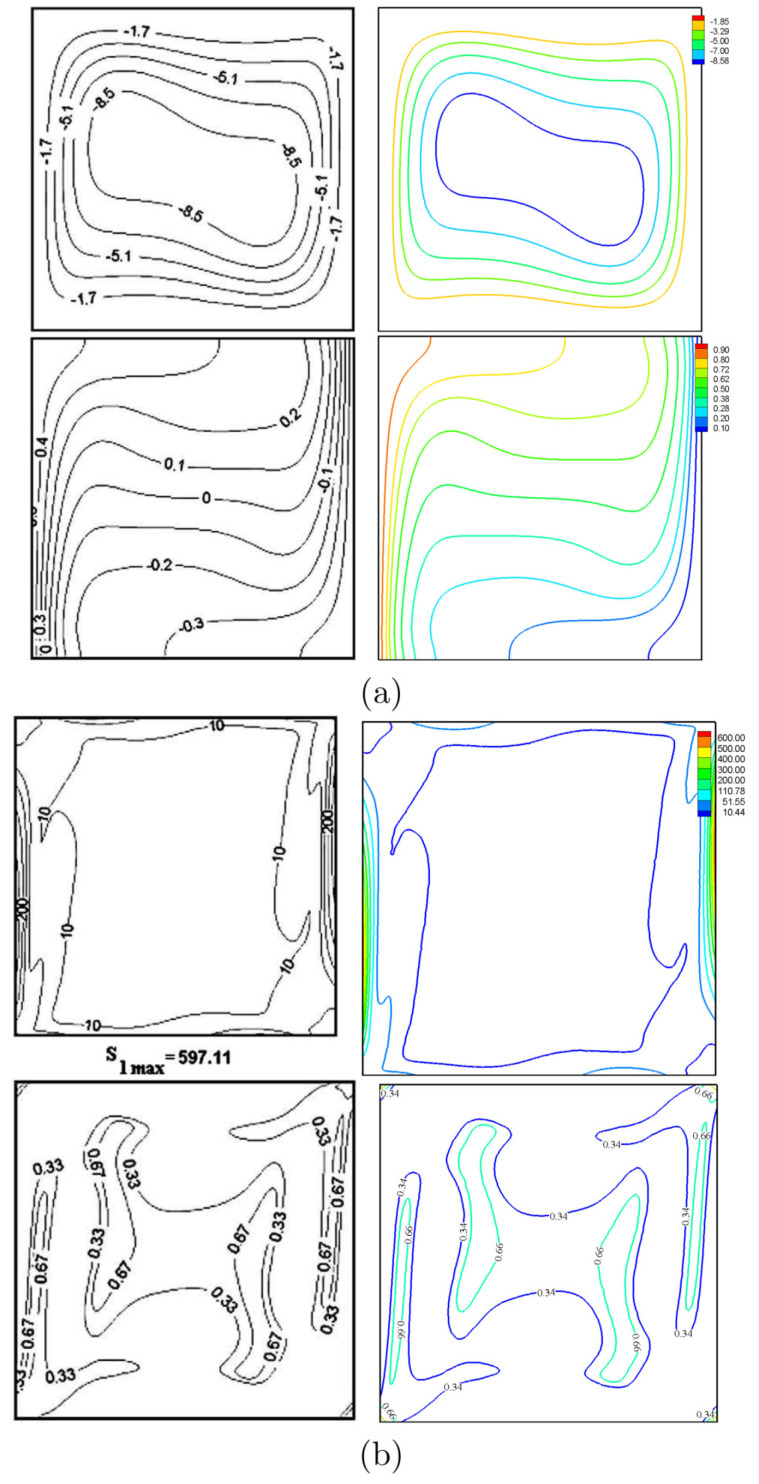
Streamlines and isotherms (**a**); global entropy generation and Bejan number (**b**). Ilis et al. [39] (**left**), present study (**right**), for Ω=0, N=0, $Ra=10^{5}$ and R=0.

**Figure 4:**
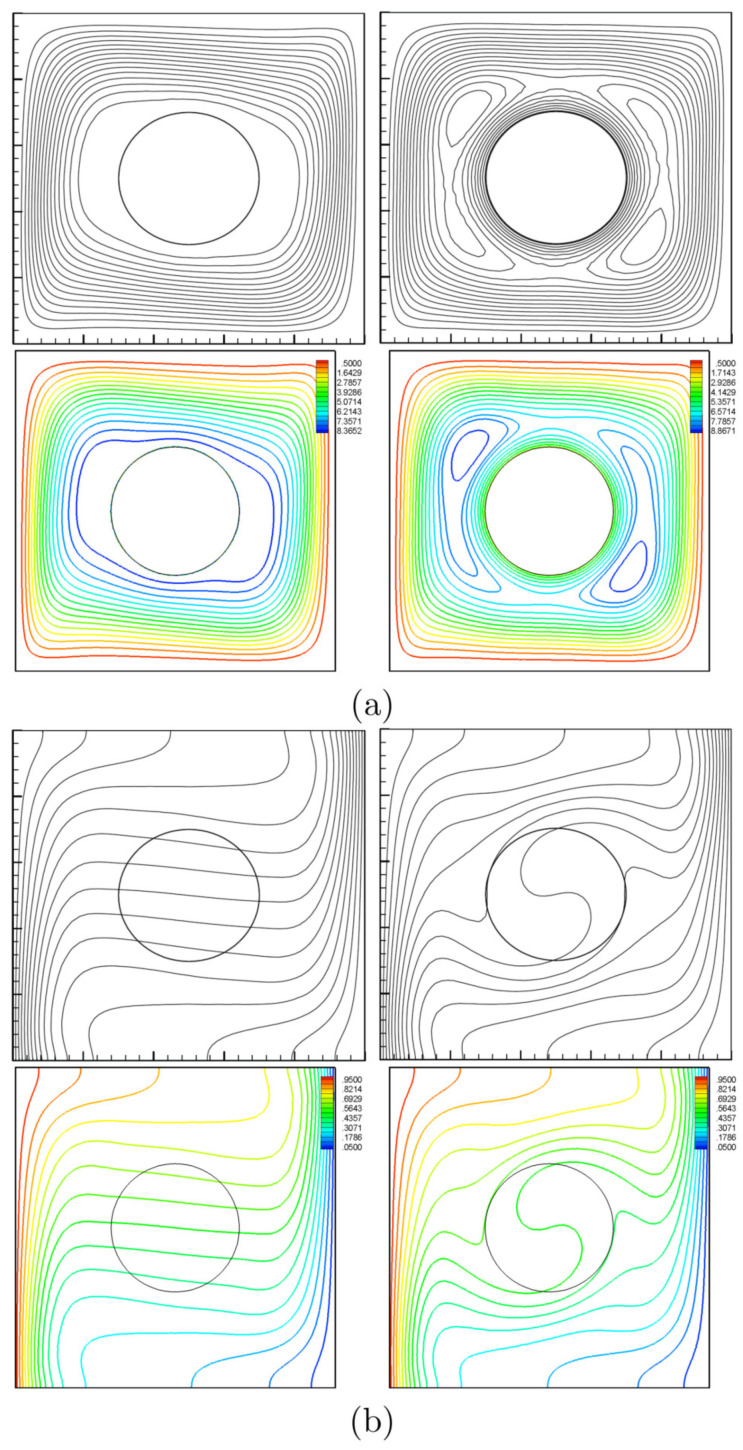
Ω=0 (**left**) and Ω=500 (**right**) for streamlines (**a**) and isotherms (**b**); Costa and Raimundo [14] (**top**) and present study (**bottom**) at N=0, $Ra=10^{5}$, $K_{r}=1$, R=0.2 and Pr=0.7.

For the sake of verifying the data, the figures of the current work are compared with the ones reported by Ilis et al. [39] for the case of entropy generation and natural convection in a square cavity heated from sides, as shown in Figure 3. In addition, a comparison is made between the resulting figures and the one provided by Costa and Raimundo [14] for the case of mixed convection in a square cavity heated from sides and with a solid rotating cylinder, as shown in Figure 4. These results provide confidence in the accuracy of the present numerical method.

## 4. Results and Discussion

This section explains the numerical outputs for the streamlines, isotherms, and the isentropic lines for five parameters. These are the Rayleigh number ($Ra=10^{3}-10^{6}$), angular rotational velocity (0≤Ω≤750), number of undulations (0≤N≤4), nanoparticle volume fraction (0≤ϕ≤0.04) and the dimensionless length of the heat source (0.2≤H≤0.8). The values of the irreversibility distribution ratio, amplitude, thermal conductivity of the solid cylinder (brickwork), dimensionless radius of rotating cylinder, dimensionless length of the surface of the cylinder and Prandtl number are fixed at $N_{\mu }=10^{-3}$, A=0.1, $k_{s}=0.76$ W/m·K, R=0.2, Θ=360 and Pr=4.623, respectively. The values of the local and average Nusselt numbers are also calculated for various values of Ω, Ra and ϕ. The thermo-physical properties of the base fluid (water) and the solid Al${}_{2}$O${}_{3}$ phases are tabulated in Table 2.

**Figure 5:**
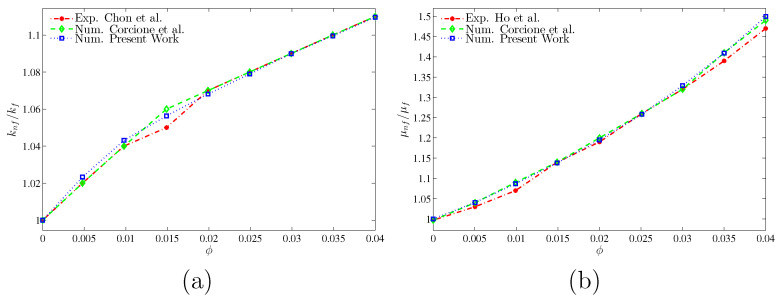
Comparison of (**a**) thermal conductivity ratio with Chon et al. [61] and Corcione et al. [62] and (**b**) dynamic viscosity ratio with Ho et al. [63] and Corcione et al. [62].

To assure the validity of the used nanofluid models of the most two effected properties namely, the thermal conductivity and the dynamic viscosity, we have conducted a comparison with other experimental data and proved models as shown in Figure 5. The results show very low discrepancies within the studied range of the volume fraction. It is convenient to show that the nanoparticles at 0.035 volume fraction enhance the thermal conductivity by 10% and raise the dynamic viscosity by about 40%. As a strategy of clarifying the effects of the studied parameters, the numerical results are presented in the following subsections.

### 4.1. Effect of the Rotational Speed

**Figure 6:**
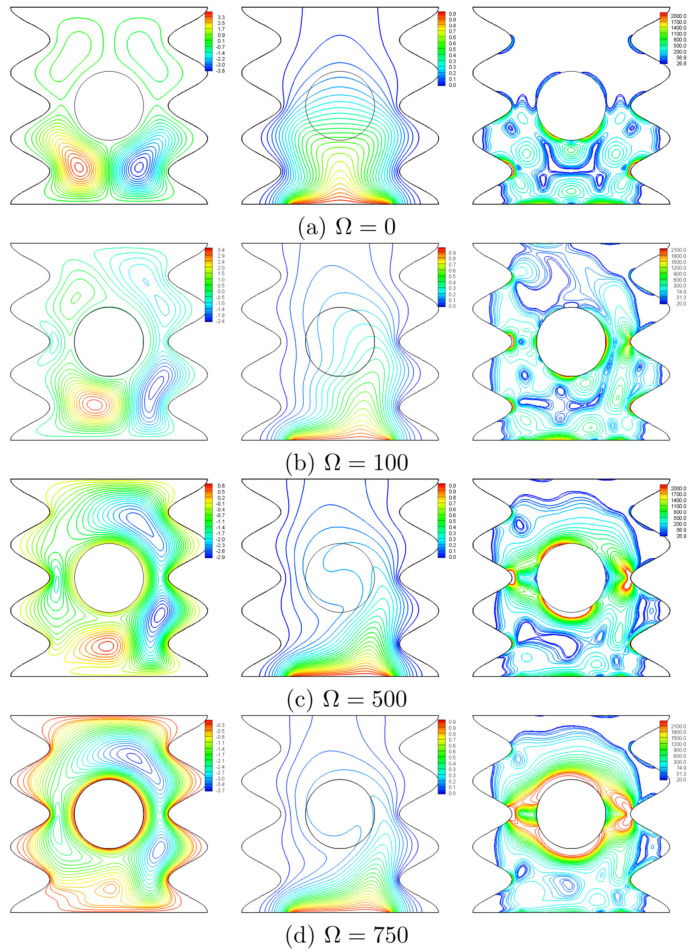
Variation of the streamlines (**left**), isotherms (**middle**), and isentropic lines (**right**) evolution by rotational speed (Ω) for N=3, $Ra=10^{5}$, ϕ=0.02 and H=0.5.

The effect of the rotational speed of the cylinder is inspected at N=3, $Ra=10^{5}$, ϕ=0.02 and H=0.5 and shown in Figure 6. Figure 6a portrays the symmetric streamlines, isotherms and the isentropic lines for a fixed cylinder (Ω=0), where it hinders the convective currents rising from the heater, thus, the nanofluid flow intensifies below the cylinder in two symmetric counter rotating vortices. The isotherms appear in a plume-like pattern and follow the geometry of lower undulations. Within the cylinder, the isotherms are approximately horizontal. A thin thermal boundary layer is seen close to the edges of the heater. Other thermal boundary layers are seen at the wall segments, which are protruded into the cavity. As a result, the isentropic lines show a concentrated entropy generation close to three regions which are the heater edges, lower undulations, and the lower surfaces of the cylinder. These entropy regions result from the heat transfer irreversibility (HTI) and the nanofluid flow irreversibility (NFI) that arise for high temperatures and velocity gradients. For a non-zero rotation, the shear action imposed by the rotating cylinder produces random streamlines with multi-cellular patterns as shown in Figure 6b–d. At Ω=100 (Figure 6b), the nanofluid follows the undulations of the right wall only, while for higher rotational speeds, the flow fills the entire cavity and follows all undulations of both walls as portrayed in Figure 6c,d. When Ω evolves, the isotherms are disturbed and follow the geometry of the lower part of the right wavy wall and their plume skews to the right. The isentropic lines show that the rotating cylinder plays a significant role in generating the entropy, where the right column of Figure 6b–d portrays the expansion of the situations of these sources. The physical cause behind this is the intensified flow within the contracted space between the ripple (protruding undulation) and the cylinder surface as obviously shown in Figure 6d.

**Figure 7:**
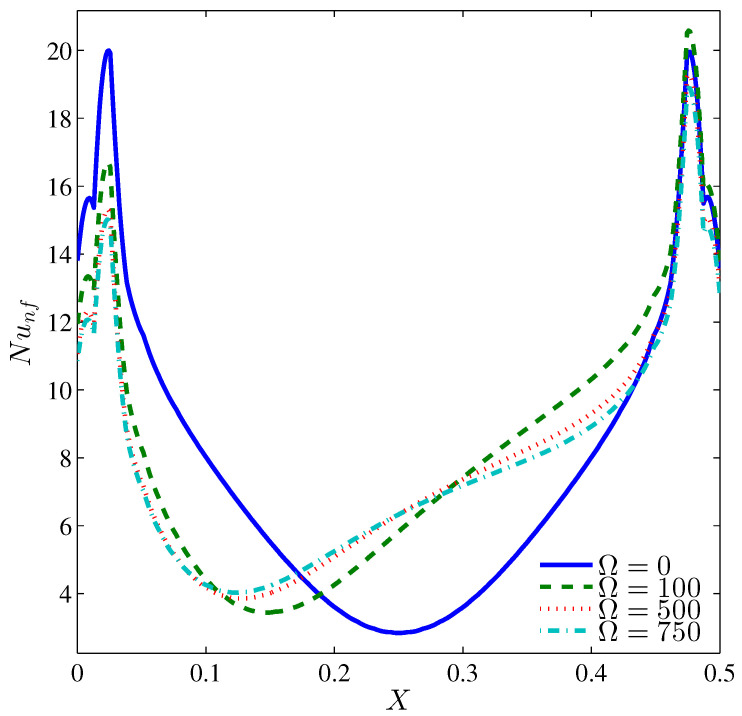
Variation of local Nusselt number interfaces with *X* for different Ω at N=3, $Ra=10^{5}$, ϕ=0.02 and H=0.5.

**Figure 8:**
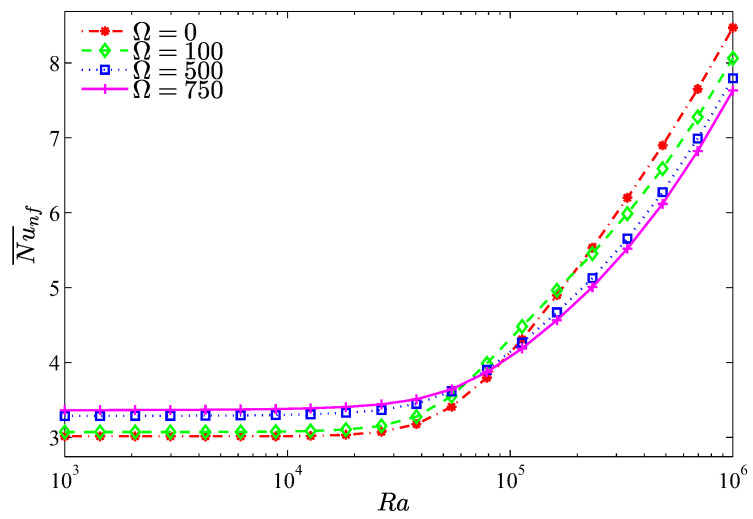
Variation of the average Nusselt number with Ra for different Ω at N=3, ϕ=0.02, H=0.5.

The distribution of the local Nusselt number, shown in Figure 7, exhibits maximum heat transfer close to heater edges while it drops at the heater center. This fashion is symmetric at Ω=0 whereas for a counter clockwise cylinder rotation, a fluid motion opposite to the rising fluid within the left part of the cavity is induced. Thus, the minimum heat exchange takes place there. Figure 8 depicts the average Nusselt number, which interprets the overall heat exchange between the heater and the nanofluid, corresponds to an aiding role of the cylinder rotation in augmenting the heat exchange when the Rayleigh number is approximately less than $8\times 10^{4}$ where the shear action of the cylinder is sufficient to guide the natural convection arising from the heater. When $Ra>8\times 10^{4}$, the natural convection strengthen and the rotating cylinder induces adversely the fluid opposite to these currents, as such, the average Nusselt number decreases with increasing values of Ω.

**Figure 9:**
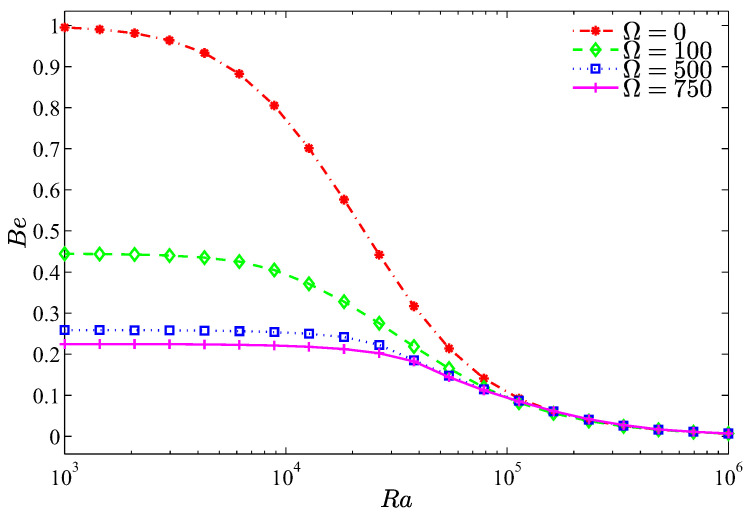
Variation of Bejan number with Ra for different Ω at N=3, ϕ=0.02, H=0.5.

Bejan number is an important parameter that shows the dominance of the heat transfer irreversibility (HTI) or the nanofluid flow irreversibility (NFI), where when Be>0.5, the HTI is dominant, while when Be<0.5, the NFI is the dominant. For $Ra<10^{5}$, Figure 9 shows how the heat transfer irreversibility dominates for a motionless cylinder, while for a non-zero cylinder rotation, the irreversibility results mainly from the nanofluid friction irreversibility (Be<0.5). The asymptotic behavior of Figure 9 indicates that beyond $Ra=10^{5}$, the strong inertia forces invigorate the friction irreversibility regardless of the cylinder speed.

### 4.2. Effect of Undulations

**Figure 10:**
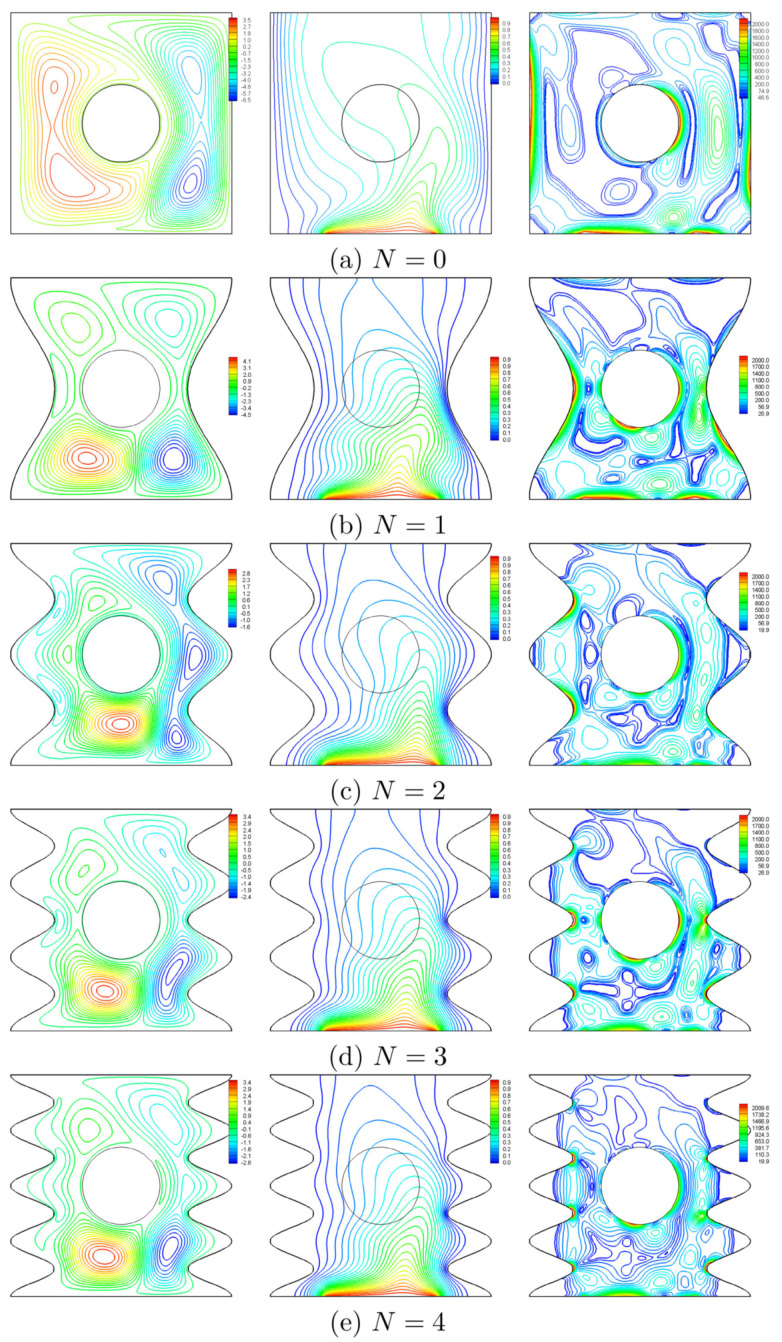
Variation of the streamlines (**left**), isotherms (**middle**), and isentropic lines (**right**) evolution by number of undulations (*N*) for Ω=250, $Ra=10^{5}$, ϕ=0.02 and H=0.5.

The impact of the number of undulations on the patterns of the nanofluid flow, isotherms and the map of entropy generation is depicted in Figure 10 for a rotational speed of 250, $Ra=10^{5}$, ϕ=0.02 and H=0.5. For flat vertical walls (N=0), Figure 10a shows the action of the rotating cylinder by contracting the counter rotating asymmetric vortices which extend within the whole cavity. This behavior was reported by Liao and Lin [16]. Besides to the main thermal boundary layer close to the heater, the plume-like isotherms skew to the right resulting in a thick boundary layer close to the right wall, whereas the entropy generates in multi-situation, especially in the right half of the cavity. For N=1, the narrow space between the cylinder and the wall segment restricts the rise up of the nanofluid, thus, the nanofluid intensifies below the cylinder in two symmetric vortices with two secondary vortices in the upper part of the cavity as shown in Figure 10b. When N=2, the space between the cylinder and the wavy wall expands, thus the nanofluid rises up freely resulting in a multi-cellular pattern of the streamlines as shown in Figure 10c. For N=3, the space mentioned above contracts again and the fluid intensifies below the cylinder as well (Figure 10d). This behavior is approximately repeated as *N* is increased to 4 as shown in Figure 10e. The isothermal maps of Figure 10 illustrate that the left valleys along with the upper right ones are completely isothermal, while the isotherms crowd at the lower protruding segments of the right wall. The isentropic lines show that the entropy is generated mainly in the lower half of the cavity and localizes at the heater edges and at the protruding segments of the wavy walls. Generally, the valleys associated with N>2 look as “idle” regions.

**Figure 11:**
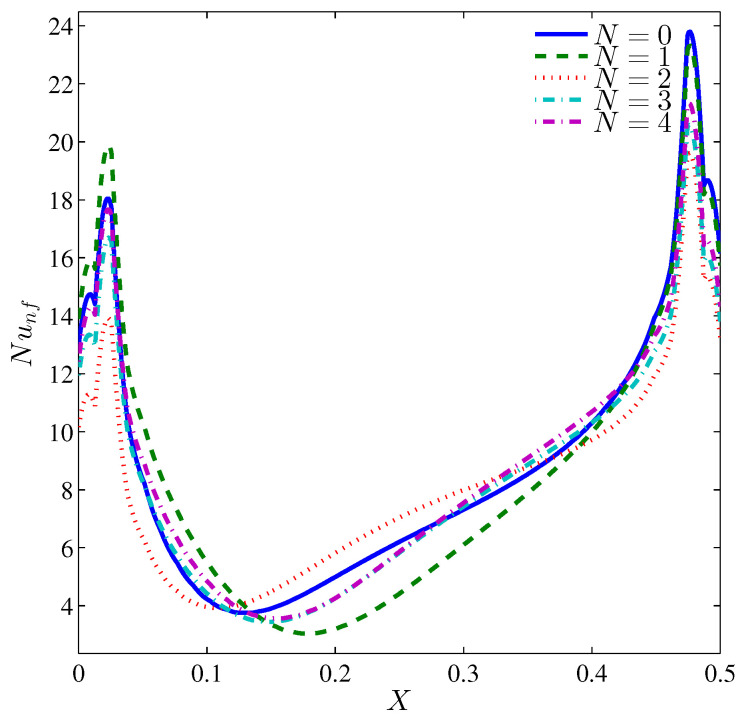
Variation of the local Nusselt number interfaces with *X* for different *N* at Ω=250, $Ra=10^{5}$, ϕ=0.02 and H=0.5.

**Figure 12:**
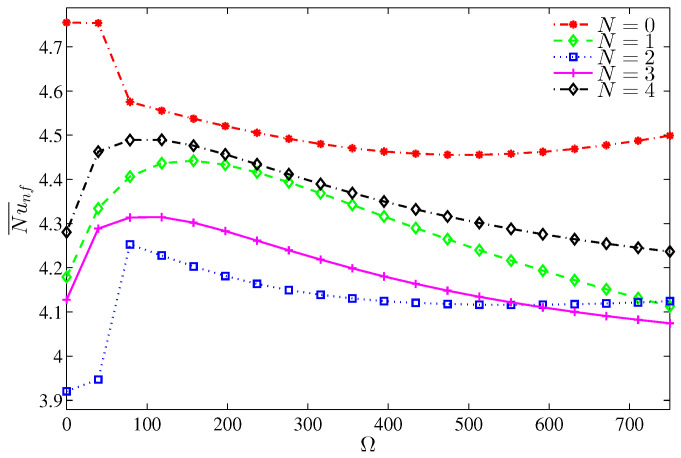
Variation of the average Nusselt number with Ω for different *N* at $Ra=10^{5}$, ϕ=0.02, H=0.5.

**Figure 13:**
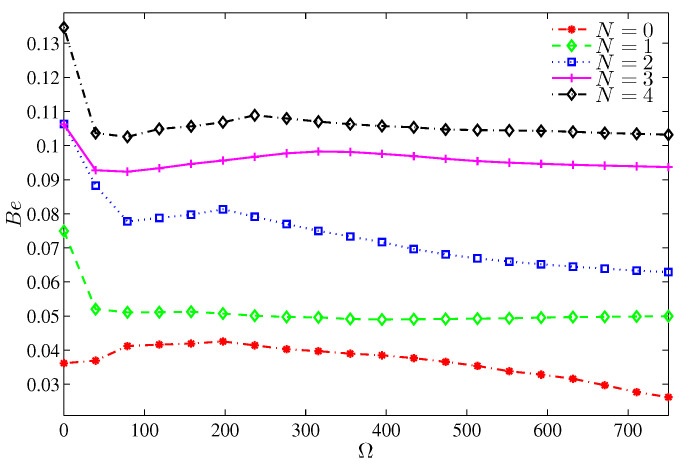
Variation of Bejan number with Ω for different *N* at $Ra=10^{5}$, ϕ=0.02, H=0.5.

The local Nusselt number varies along the heater surface in an alternative fashion with N (Figure 11). However, at the heater center, the local Nusselt number is maximum for N=2 and minimum for N=3. This is because the available space between the rotating cylinder and a segment of the wavy wall is maximum and minimum, respectively. The average Nusselt number, Figure 12, is obviously influenced by the number of undulation for a still cylinder, while for a rotating cylinder, the influence of *N* dwindles. It should be noticed that at $Ra=10^{5}$, the existence of the undulation in the cold walls reduces the average Nusselt number despite the available cold area by which the heat is dissipated. The reason of this reduction refers to the drag effect of the protruding segments of the wavy wall and the isothermal valleys, which act as dead zones at relatively low Rayleigh numbers. When N=2, the worst Nusselt number, associated with the single large vortex standing on the heater surface, is found and restricts the rise of energy in turn. On the other hand, with four undulations (N=4), the substantial cold area of the wavy wall helps in better values of the Nusselt number. Because the flow separates early from the lower protruding of the wavy wall, the Bejan number increases with the number of undulations (Figure 13) which means the decrease of NFI.

### 4.3. Effect of Nanofluid Loading

**Figure 14:**
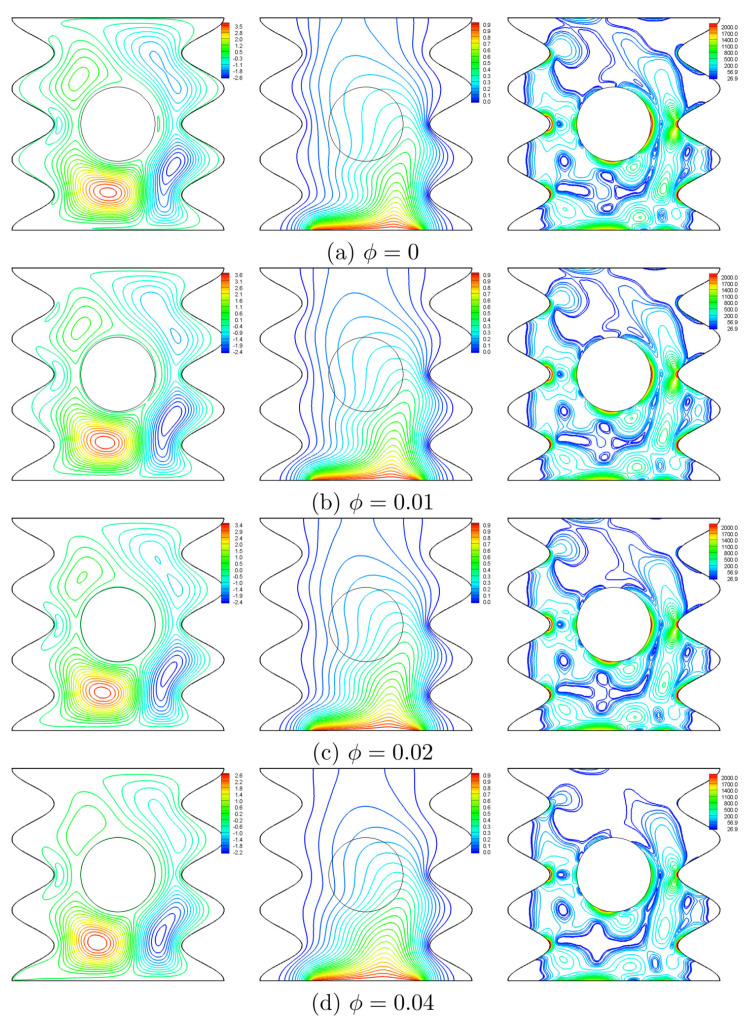
Variation of the streamlines (**left**), isotherms (**middle**), and isentropic lines (**right**) evolution by the volume fraction (ϕ) for Ω=250, N=3, $Ra=10^{5}$ and H=0.5.

**Figure 15:**
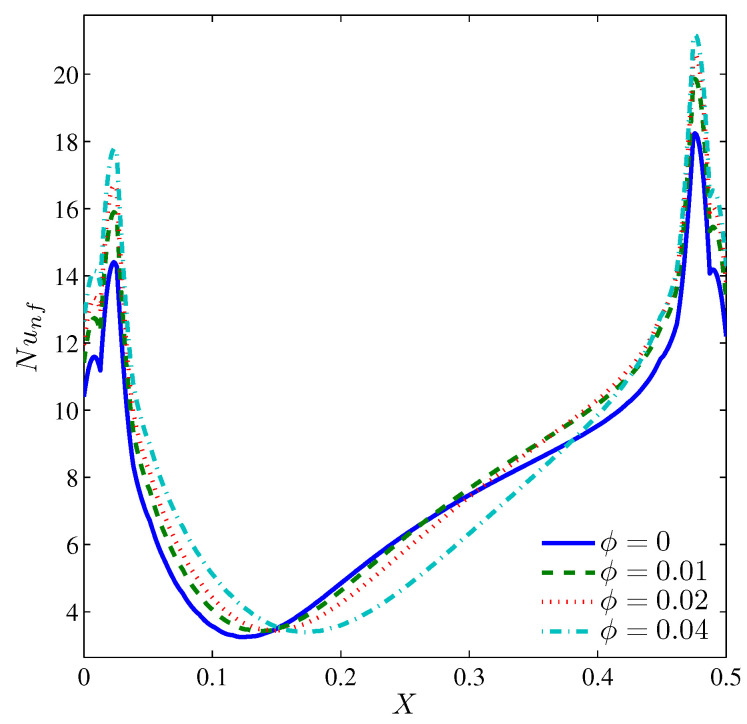
Variation of the local Nusselt number interfaces with *X* for different ϕ at Ω=250, N=3, $Ra=10^{5}$ and H=0.5.

In this subsection, we will discuss the effect of the nanoparticles volume fraction for N=3, $Ra=10^{5}$, and H=0.5. From Figure 14, it is evident that increasing the volume fraction of the nanoparticles does not greatly affect the isotherms and the isentropic lines. Because of the growth of the viscous force with increasing ϕ, the streamlines depict that the intensity of flow weakens and drifts apart from the valleys especially those on the left. The distribution of the local Nusselt number (Figure 15) shows a local heat transfer enhancement with increasing values of ϕ, except at the center of the heater where the two counter rotating vortices are met, thus, the rising convective currents are blocked; and as such the viscous and inertia forces gained by ϕ overcome the enhancement of the thermal conductivity.

**Figure 16:**
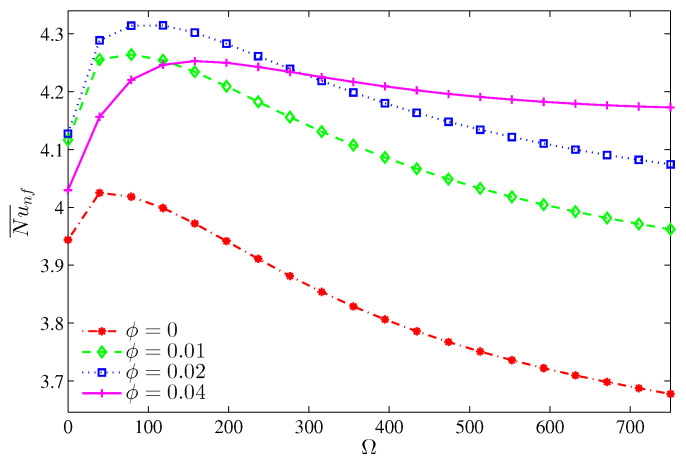
Variation of the average Nusselt number with Ω for different ϕ at N=3, $Ra=10^{5}$, H=0.5.

**Figure 17:**
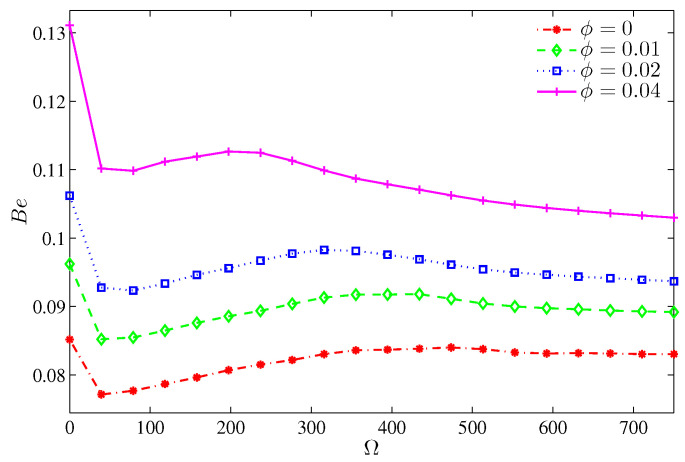
Variation of Bejan number with Ω for different ϕ at N=3, $Ra=10^{5}$, H=0.5.

The variation of the average Nusselt number shown in Figure 16 demonstrates an enhancement of the overall heat transfer with ϕ. It should be noted that an extra loading of nanoparticles for a still cylinder and even when Ω≤300 deteriorates the overall heat transfer, which corresponds to the dominance of the viscous and inertia forces, thus, the average Nusselt number drops within this range of rotation. Figure 17 illustrates the dominance of the NFI. However, by increasing the nanoparticles volume fraction, the HTI tends to be slightly effective. This can be imputed to the increase in the thermal conductivity, which transports more heat energy. However, the rotation of the cylinder induces more NFI, thus, beyond Ω=0, the Bejan number decays rapidly.

### 4.4. Effect of Heater Length

**Figure 18:**
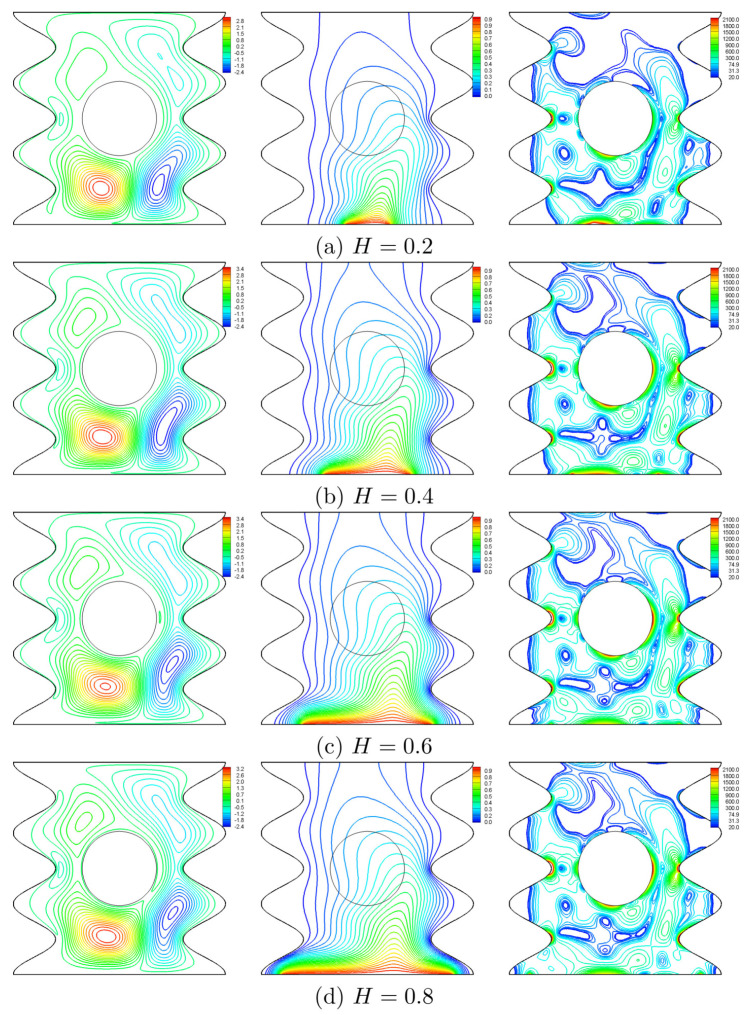
Variation of the streamlines (**left**), isotherms (**middle**), and isentropic lines (**right**) evolution by the heater length (*H*) for Ω=250, N=3, $Ra=10^{5}$ and ϕ=0.02.

**Figure 19:**
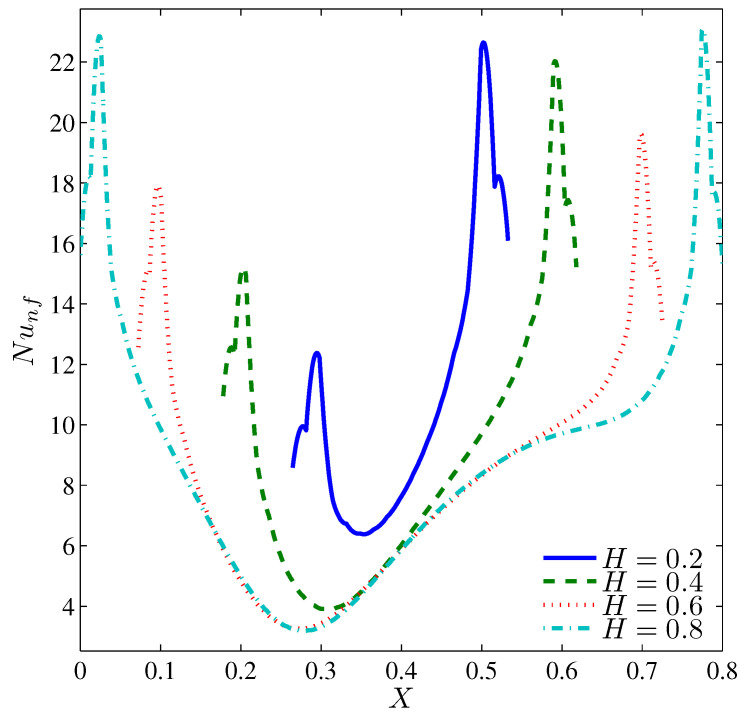
Variation of the local Nusselt number interfaces with *X* for different *H* at Ω=250, N=3, $Ra=10^{5}$ and ϕ=0.02.

**Figure 20:**
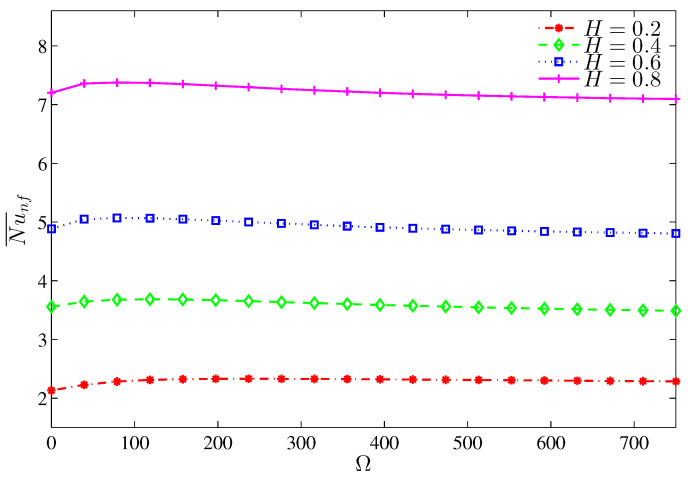
Variation of the average Nusselt number with Ω for different *H* at N=3, $Ra=10^{5}$ and ϕ=0.02.

**Figure 21:**
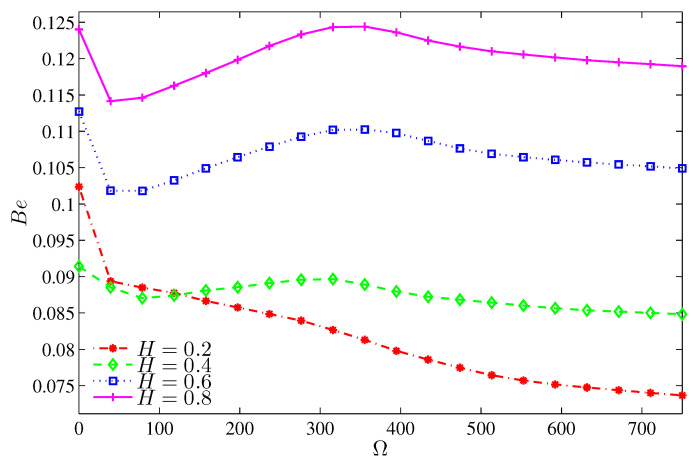
Variation of Bejan number with Ω for different *H* at N=3, $Ra=10^{5}$ and ϕ=0.02.

Figure 18, Figure 19, Figure 20 and Figure 21 illustrate the effect of the heater length on the nanofluid flow, thermal fields, and the isentropic lines. The contour maps (Figure 18) show no significant variation in the streamlines with increasing values of *H*, while the isotherms show the extension of the thermal boundary layer along the heater surface. The isentropic lines depict the expansion of the entropy sources to occupy substantial regions of the lower valleys. Figure 19 shows that the local heat exchange is less for a larger heater size. This refers to the fact that the nanofluid circulation carries more energy when passes along a substantially hot region, so it will be hotter; and as such, the local Nusselt number decreases. Alternatively, the overall heat exchange increases with increasing values of *H* (Figure 20) because large quantities of the circulating nanofluid contribute in the heat exchange process. The pattern of the entropy generation rate depicts an increasing behavior with increasing values of *H* except at Ω=0, where the entropy generation rate owing to HTI at H=0.2 is greater than what is generated at H=0.4. Approximately, the Bejan number is constant with Ω for H≥0.4 whereas, at H=0.2, the Bejan number decreases with Ω as shown in Figure 21. This trend can be attributed to the fact that the radius of the rotating cylinder corresponds to this heater size, thus, the rotation action of the cylinder contributes in destroying the thermal boundary layer which is concentrated at the heater edges which in turn, decreases the HTI.

## 5. Conclusions

This paper presents a numerical study of entropy generation in mixed convection resulting from the rotation of a conductive cylinder inside a cavity with wavy walls under the assumption of laminar 2D flow. An alumina–water nanofluid fills the cavity. Based on the results above, we can draw the following conclusions:

1. The role of the cylinder velocity is related to other parameters in a sophisticated manner. Based on the heat transfer enhancement, we found that for a wavy wall (N≠0) and for $Ra\leq 10^{5}$, the optimum velocity should be within the range 100–200.
2. For $Ra\geq 10^{5}$, the rotation of the cylinder exhibits an adverse action on the convective heat transfer.
3. For a motionless cylinder, the heat transfer irreversibility is dominant, while for a rotating cylinder, the generated entropy results mainly from the nanofluid friction irreversibility.
4. The entropy generation due to the nanofluid friction irreversibility increases with increasing values of the rotational velocity within the Rayleigh range of $Ra\leq 10^{5}$, while beyond this range, the rotational speed has no effect on the irreversibility ratio.
5. The entropy generation due to heat transfer irreversibility becomes significant when the number of undulation increases; this is associated with the early separation of nanofluid from the lower parts of the wavy wall.
6. The number of undulations affects the average Nusselt number for a still cylinder, while for a rotating cylinder, this effect is less significant. However, at $Ra=10^{5}$, the undulation of the walls reduces the average Nusselt number.
7. The convective heat transfer rises with the increase of the volume fraction of the nanoparticles and with the length of the heater segment.
8. The Bejan number increases with the increase of the number of undulations because of the early separation from the lower protruding segment.
9. When the heater size corresponds to the radius of the rotating cylinder, the thermal boundary layer, which is concentrated at the heater edges, is destroyed.

## Figures and Tables

**Table 1:** Grid testing for $\Psi_{\min}$, ${\bar{Nu}}_{nf}$ and Be at different grid sizes for Ω=250, N=3, $Ra=10^{5}$, ϕ=0.02 and H=0.5.

Grid Size	Number of Elements	$\Psi_{\min}$	${\bar{\mathrm{Nu}}}_{\mathrm{nf}}$	Be
G1	2971	−2.5253	3.9993	0.096561
G2	3403	−2.5422	4.0531	0.096649
G3	3909	−2.5707	4.0668	0.096811
G4	4810	−2.5901	4.1051	0.097009
G5	11,794	−2.6276	4.2542	0.097107
**G6**	27,151	−2.6407	4.2552	0.097162
G7	32,745	−2.6443	4.2562	0.097179

**Table 2:** Thermo-physical properties of water with Al${}_{2}$O${}_{3}$ nanoparticles at T=310 K [60].

Physical Properties	Fluid Phase (Water)	Al${}_{2}$O${}_{3}$
$C_{p}\;\left(J/\mathrm{kgK}\right)$	4178	765
$\rho \;(\mathrm{kg}/m^{3})$	993	3970
$k\;({\mathrm{Wm}}^{-1}K^{-1})$	0.628	40
$\beta \times 10^{5}\;\left(1/K\right)$	36.2	0.85
$\mu \times 10^{6}\;\left(\mathrm{kg}/\mathrm{ms}\right)$	695	–
$d_{p}\;\left(\mathrm{nm}\right)$	0.385	33

## Abbreviations

The following abbreviations are used in this manuscript:

*A*	amplitude
Be	Bejan number
$C_{p}$	specific heat capacity
$d_{f}$	diameter of the base fluid molecule
$d_{p}$	diameter of the nanoparticle
*g*	gravitational acceleration
GEG	Dimensionless global entropy generation
*H*	dimensionless length of the heat source, H=h/L
*k*	thermal conductivity
$K_{r}$	inner block to nanofluid thermal conductivity ratio, $K_{r}=k_{s}/k_{nf}$
*L*	width and height of the square cavity
*N*	number of undulations
$N_{\mu }$	irreversibility distribution ratio
$\bar{Nu}$	average Nusselt number
Pr	Prandtl number
*r* & *R*	radius of rotating cylinder & dimensionless radius of rotating cylinder
Ra	Rayleigh number
$S_{\mathrm{gen}}$	Entropy generation rate
$S_{\mathrm{GEN}}$	Dimensionless entropy generation rate
$S_{\theta }$	dimensionless entropy generation due to heat transfer irreversibility
$S_{\Psi }$	dimensionless entropy generation nanofluid friction irreversibility
ReB	Brownian motion Reynolds number
*T*	temperature
$T_{0}$	reference temperature (310 K)
$T_{fr}$	freezing point of the base fluid (273.15 K)
v, V	velocity and dimensionless velocity vector
$u_{B}$	Brownian velocity of the nanoparticle
*x*, *y* & *X*, *Y*	space coordinates & dimensionless space coordinates
Greek symbols	
α	thermal diffusivity
β	thermal expansion coefficient
δ	normalized temperature parameter
θ	dimensionless temperature
Θ	dimensionless length of the surface of the cylinder
μ	dynamic viscosity
ν	kinematic viscosity
ρ	density
ϕ	solid volume fraction
ω,Ω	angular rotational velocity, dimensionless angular rotational velocity
Subscript	
*b*	bottom
*c*	cold
*f*	base fluid
*h*	hot
nf	nanofluid
*p*	solid nanoparticles
*s*	solid cylinder
